# Improved Selective BIN Agar for a Better Rate of Yersinia pestis Isolation from Primary Clinical Specimens in Suspected Madagascar Plague Cases

**DOI:** 10.1128/JCM.00564-21

**Published:** 2021-07-19

**Authors:** Moshe Aftalion, Ronit Aloni-Grinstein, Voahangy Andrianaivoarimanana, Alice Lantoniaina Iharisoa, Shlomo Shmaya, David Gur, Orly Laskar, Minoarisoa Rajerison, Emanuelle Mamroud

**Affiliations:** a Department of Biochemistry and Molecular Genetics, Israel Institute for Biological Research, Ness-Ziona, Israel; b Plague Unit, Central Laboratory for Plague, Institut Pasteur de Madagascar, Antananarivo, Madagascar; c Department of Infectious diseases, Israel Institute for Biological Research, Ness-Ziona, Israel; Medical College of Wisconsin

**Keywords:** Madagascar, *Yersinia pestis*, clinical samples, diagnostics, plague, selective medium

## Abstract

According to the WHO, 75% of the world’s plague cases are found in Madagascar, with an average of 200 to 700 cases suspected annually (mainly bubonic plague). In 2017, a pneumonic plague epidemic of unusual proportions occurred, which raised several challenges for laboratory confirmation of cases, pointing to the need for the development of Yersinia pestis isolation procedures, especially those that can be performed in remote areas. As the WHO gold standard for plague diagnosis is bacterial culture, we sought to develop a simple method to prepare a highly selective medium, fit for use in remote areas where plague is endemic. The performance of the new medium, named improved BIN, was examined in terms of growth support and selectivity with spiked samples as well in isolating Y. pestis from clinical specimens, and it was compared to the results obtained with commercially available selective media. The preparation of the new medium is less complex and its performance was found to be superior to that of first-generation BIN medium. The growth support of the medium is higher, there is no batch diversity, and it maintains high selectivity properties. In 55 clinical specimens obtained from patients suspected to be infected with Y. pestis, approximately 20% more Y. pestis-positive isolates were identified by the improved BIN medium than were identified by commercially available selective media. The improved BIN medium is notably advantageous for the isolation of Y. pestis from clinical specimens obtained from plague patients, thus offering better surveillance tools and proper promotion of medical treatment to more patients suspected of being infected with Y. pestis.

## INTRODUCTION

Yersinia pestis, the causative agent of the plague, is designated by the CDC as a tier 1 agent, mainly because of rapid progression and severity of the disease and the person-to-person transmission rate (1). Recent outbreaks have been documented in Uganda (2), China (3), the Democratic Republic of Congo (4), and Madagascar (5). High mortality rates occur if treatment is not initiated within 18 to 24 h of the onset of symptoms. Hence, prompt identification of the causative agent is mandatory. Many sophisticated molecular, immunological, and biochemical approaches have been implemented for pathogen identification; however, these techniques are not feasible for use in remote locations around the world where plague is endemic. Moreover, according to the World Health Organization, bacteriological culture with Y. pestis strain isolation remains the gold standard for a plague diagnosis (6). Here, we describe a new, improved, and simple protocol for producing selective BIN agar plates for the isolation of Y. pestis from various contaminated clinical and environmental samples. The improved BIN agar plates allow the isolation of clinical Y. pestis isolates in Madagascar that were not isolated by other means.

## MATERIALS AND METHODS

### Media, agar plates, selective supplements, and bacterial strains.

The formulation and preparation of first-generation BIN, selective solid agar medium supplemented with cefsulodin-Irgasan-novobiocin (CIN), MacConkey agar and Luria-Bertani broth (BHI), Y. pestis (Table 1), Escherichia coli ATCC 25922, and Bacillus anthracis Δ14185 strains are all described in detail in Ber et al. (7). All supplements used are described in Ber et al. (7), except for gentian violet (catalog no. G-2039-25G; Sigma).

**TABLE 1:** Y. pestis strains

Yersinia pestis strain	Relevant characteristics	Reference
Kimberley53 ΔpCD1 ΔpPCP1	Spontaneously pPCP1 and pCD1-cured Kimberley53	16
EV76 (pGFPuv)	*pgm*^−^ (Girard’s strain)	7
A1122	pCD1 cured	17

### Improved BIN agar plates.

BHI agar (BHIA) was prepared and boiled (100°C) for 1 min according to the manufacturer’s instructions, omitting the autoclave step. Upon cooling to ∼50°C, supplements in an all-in-one stock solution were added to the boiled BHI agar. The all-in-one stock solution was prepared as follows: 0.08g of Irgasan (triclosan, catalog no. GA5716; Glentham Life Sciences, UK) was dissolved in 10 ml of 90% ethanol absolute (catalog no. 1009832500; Merck) to a final concentration of 8 mg/ml. A 10 mg/ml gentian violet solution (catalog no. G-2039-25G; Sigma) was prepared by dissolving 0.5 g in 50 ml double-distilled water (ddH_2_O). The solution was sterilized by autoclaving. Five grams of Na-cholate (catalog no. C-9282; Sigma) and 5 g of Na-deoxycholate (catalog no. D-6750; Sigma) were dissolved in 90 ml of ddH_2_O to a final concentration of 55.56 mg each/ml. Immediately after autoclave sterilization, 1 ml of Irgasan was added to the cholate solution, and the solution was homogenized vigorously for 1 min. Following cooling to room temperature, 1 ml of the gentian violet solution was added and mixed. Then, 322 mg of nystatin (catalog no. N3503-25MU; Sigma) was added and mixed to the final all-in-one stock solution. The all-in-one solution may be stored at 4°C for at least 1 year.

### Plating efficiency, growth rates, and selective tests.

Plating efficiency tests, evaluation of growth rates on semisolid agar, and selective tests are described in detail in Ber et al. (7).

### Environmental sample collection.

Environmental samples were collected from different areas in Israel and represent vast microbial content, including *Bacillus cereus*, *Bacillus megaterium*, *Bacillus thuringensis*, *Bacillus subtilis*, *Erwinia herbicola*, and *Pseudomonas aeruginosa*, as described in Aloni-Grinstein et al. (8).

### Biological plague sample collection and plague diagnosis as per the Plague National Control Programme.

Human biological specimens (bubo aspirate, sputum samples, or postmortem organ puncture specimens) used in this study were collected by the Central Laboratory for Plague (CLP) and the Institute Pasteur de Madagascar (IPM) as part of the Plague National Control Programme (PNCP) of the Malagasy Ministry of Public Health. The PNCP requires the declaration of all suspected human plague cases on a standardized notification form and collection of biological specimens on sterile swabs conveyed in Cary-Blair transport medium. These specimens were collected under this mandatory reporting system and thus are considered exempt from human subject research protocols. All biological specimens were delinked from the patients’ identifiable information and analyzed anonymously. Therefore, no approval from the Malagasy Ethical Committee was required for this study. Once received at the CLP-IPM, each sample was tested by F1RDT for F1 antigen detection (9) and subjected to quantitative real-time PCR (qPCR) of two Y. pestis targets (10) and bacteriological diagnosis for Y. pestis isolation (11).

A total of 55 biological specimens from patients suspected to have plague were used for this study either retrospectively (*n* = 29) or prospectively (*n* = 26) (Table 2). For the retrospective study, 29 archived biological specimens from suspected plague patients were selected among confirmed plague cases (F1RDT and qPCR positive) without Y. pestis strain isolation (culture negative). To avoid long-term preservation issues, only specimens received within 1 year (16 October 2018 to 26 February 2019) before the evaluation were used. The chosen specimens consisted of 23 bubo aspirates, 1 sputum sample, and 5 organ puncture specimens (2 lungs and 3 livers). Archived specimens stored in Cary-Blair medium at room temperature were reextracted with 1 ml sterile phosphate-buffered saline (PBS). After homogenization, the samples were streaked on BIN medium and incubated at 26°C to 28°C for at least 48 h. BIN analysis results were compared with those of CIN that are already available in the CLP database.

**TABLE 2:** Distribution of plague biological specimens tested on BIN medium

Sample type	No. of specimens		
	Retrospective study	Prospective study	Total
Bubo	23	22	45
Sputum	1	1	2
Organ puncture	5	3	8
Total	29	26	55

The prospective study included a total of 26 biological specimens from suspected plague patients, consisting of 22 bubo aspirates, 1 sputum sample, and 3 postmortem specimens (1 lung puncture, 1 liver puncture, and 1 not specified). These specimens, received at the CLP between 8 January 2019 and 3 April 2019, were streaked on both selective CIN agar medium (Oxoid Ltd., UK) and BIN medium for comparison of Y. pestis isolation. CIN and BIN plates were incubated at 26°C to 28°C for at least 48 h.

For both studies, suspected Y. pestis colonies were identified by phage lysis tests and biochemical identification on API20E strips (bioMérieux, France).

## RESULTS

### Omitting autoclaving improves growth support and batch uniformity without impairing sterility.

**FIG 1:**
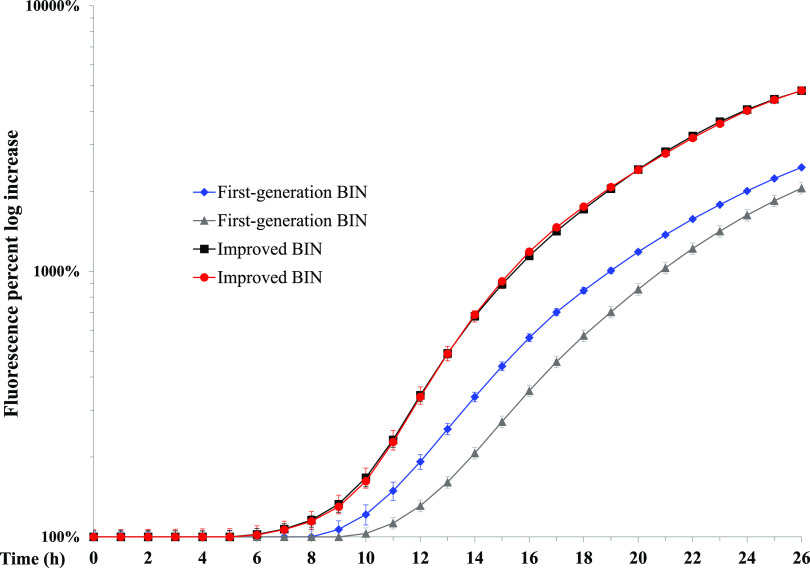
Exclusion of the autoclave steps improves growth support and uniformity of BIN medium. Thirty microliters of 10^8^ CFU/ml bacterial cultures of EV76 (pGFPuv) were plated in quadruplicate in 24-well microplates with first-generation BIN (gray and blue lines) and improved BIN (red and black lines) prepared with two batches of BHIA (batch 1, red and gray; batch 2, black and blue). The plates were incubated at 28°C in a spectrofluorometer, and the fluorescence emitted by the bacteria was determined hourly. The fluorescence percent log increase was determined by using the fluorescence values at the beginning of the incubation as background.

The optimal selective medium should balance growth support and selectivity. BIN, a selective medium for Y. pestis, was shown to be advantageous over commercial MacConkey and CIN selective media (7). However, the variability in different batches of BHIA used for BIN preparation (Fig. 1, first-generation BIN) together with the reduction in growth support compared to that obtained by nutrient-rich BHIA (7) were challenges to overcome in producing the improved medium. We found that omitting the autoclave step while retaining the 1-min boiling step allowed medium ingredients to dissolve better. Strikingly, the BHIA batch diversity seen in the first-generation BIN plates was abolished following the elimination of the autoclave step, and the growth support capacity and sterility of the plates remained high (Fig. 1, improved BIN). No contamination was found when 80 BIN plates prepared from BHIA without an autoclaving step were incubated for 7 days at room temperature. Additionally, incubation of BIN plates seeded with a standard 1-µl loop of concentrated colony suspensions (>10^9^ CFU/ml) of E. coli (a representative Gram-negative bacterium) or Bacillus anthracis Δ14185 (a representative Gram-positive bacterium) for 24 h at 37°C did not yield any bacterial growth. In summary, exclusion of the autoclave step was shown to provide better growth support and uniformity, with no need for BHIA batch growth support quality determination.

### All-in-one supplement solution.

**FIG 2:**
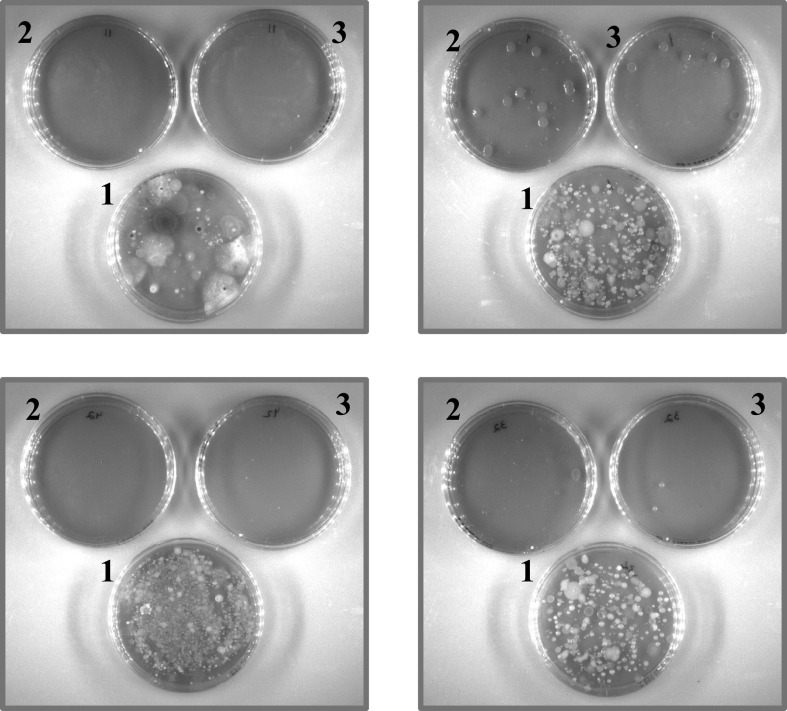
Improved BIN offers selectivity. Environmental samples (100 ml) taken from 4 different locations in Israel were plated on BHIA plates (1), first-generation BIN plates (2), and improved BIN plates (3). The plates were incubated for 24 h at 37°C and visually monitored by the unaided eye for growth.

**FIG 3:**
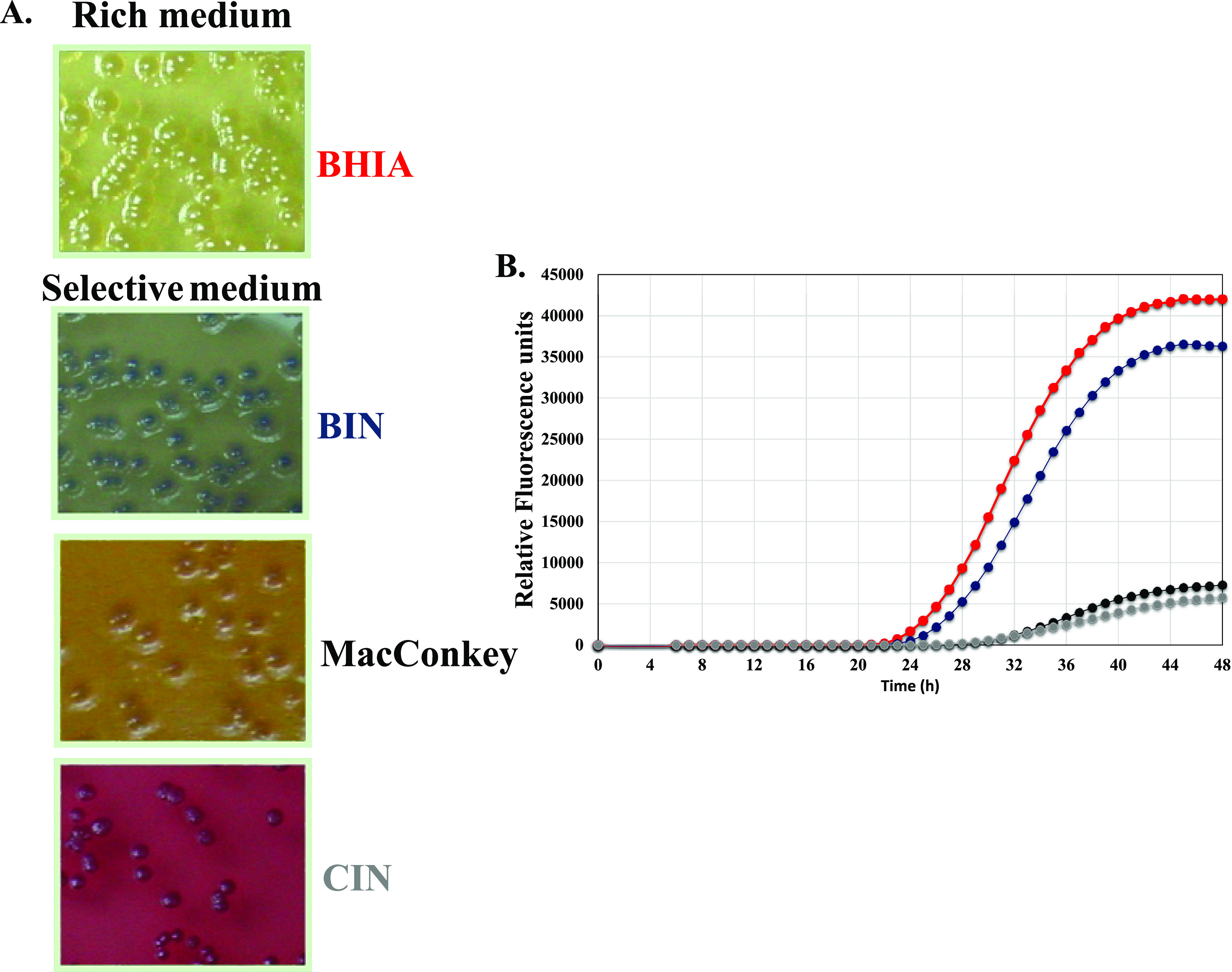
Improved BIN plates offer better growth support than the standard selective MacConkey plates and CIN plates. (A) Thirty microliters of 10^7^ CFU/ml bacterial cultures of EV76 (pGFPuv) were plated in triplicate in 24-well microplates. The plates were incubated at 28°C for 48 h and visually monitored by the unaided eye for growth. (B) Thirty microliters of 10^7^ CFU/ml bacterial cultures of EV76 (pGFPuv) were plated in quadruplicate in 24-well microplates. The plates were incubated at 28°C for 48 h in a spectrofluorometer, and the fluorescence emitted by the bacteria was determined hourly. Relative florescence units (RFU) were determined by using the fluorescence values at the beginning of the incubation as background. Red, BHIA; blue, BIN; black: MacConkey; gray, CIN.

**FIG 4:**
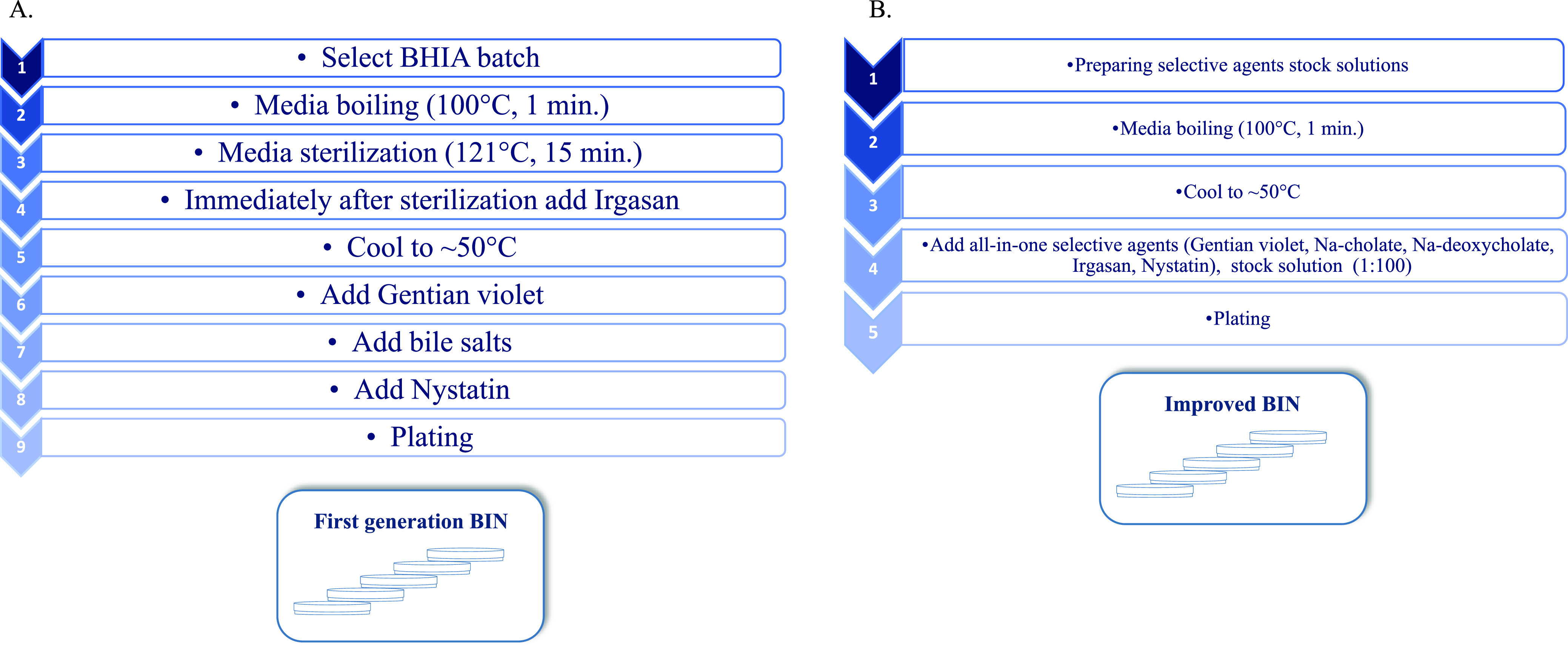
Schematic representation of the working steps required for first-generation BIN plates (A) versus improved BIN plates (B).

The addition of selective supplements to BHIA is a complex multistep procedure. The supplements included four ingredients that were stored at different temperatures. Moreover, Irgasan was dissolved in 90% ethanol, while the other supplements were dissolved in water. Each selective supplement was differentially diluted in the medium. A preprepared all-in-one supplement solution may greatly ease the preparation of BIN plates. Thus, we prepared an all-in-one supplement stock solution (improved BIN) and compared its growth support to that obtained when the supplements were added in a stepwise fashion (first-generation BIN). Using an all-in-one solution containing all the selective materials did not change the growth support of the medium for Y. pestis (data not shown). Most importantly, BIN plates prepared with the all-in-one solution have the same selective features against environmental contaminants as the BIN plates that were prepared in a sequential manner (Fig. 2), which were not evident in the nonselective BHIA plates. These results show that the improved BIN plates are advantageous over the selective CIN and MacConkey plates (Fig. 3) and offer selectivity, which is not obtained with rich BHIA plates (Fig. 2). The simplicity of the procedure for preparing the improved BIN plates, compared to the first-generation BIN plates (Fig. 4), is appealing with respect to the locations where plague is endemic, some of which do not have well-equipped facilities. The preprepared all-in-all solution may decrease human errors that may occur during serial addition of the individual supplements to the medium. Furthermore, omitting the autoclave step greatly improved the growth support of Y. pestis strains EV76 (pGFPuv), A1122, and Kimberley53 ΔpPCP1 ΔpCD1. Thus, improved BIN was chosen for further evaluation of bacterial rescue from clinical specimens.

### Improved BIN plates offer better isolation rates of Y. pestis from clinical specimens than CIN selective medium.

As prompt isolation and identification of Y. pestis from clinical specimens of potentially infected patients is crucial for proper life-saving treatment, we next evaluated the beneficial isolation features of the improved BIN medium over those obtained by CIN, the standard Y. pestis isolation medium used in Madagascar. Of note, MacConkey medium was not used for this evaluation due to its low performance compared to that of the BIN formulation (see reference 7 and Fig. 3) and the limited number of clinical specimens. For the retrospective study, among 29 specimens positive for both F1 RDT and qPCR but negative for culture using CIN medium, 3 (1 bubo, 1 lung puncture, and 1 liver puncture) yielded Y. pestis isolates on improved BIN medium. For the prospective study, 26 specimens were run in parallel on CIN medium and on the improved BIN medium (Table 3). Among the 18 specimens that tested negative on the CIN medium used routinely at the CLP, Y. pestis was isolated from 5 specimens (4 buboes and 1 lung puncture) using BIN medium. The two media showed a concordance of 80.8% (kappa, 0.62; 95% confidence internal [95% CI], 0.34 to 0.90). Among the improved BIN media that allowed the recovery of 8 Y. pestis isolates from clinical specimens, 5 exhibited pure colonies of Y. pestis, while CIN medium showed only contaminants. In summary, improved BIN plates offer better isolation properties for Y. pestis obtained from clinical specimens and greatly reduced the presence of contaminants compared to the commonly used CIN plates. Therefore, prompt and targeted medical care may be offered to more plague patients.

**TABLE 3:** Comparison of Y. pestis isolation from clinical specimens between CIN and BIN media

BIN culture result	CIN culture result (no.)		
	Positive	Negative	Total
No. positive	8	5	13
No. negative	0	13	13
Total no.	8	18	26

## DISCUSSION

Early diagnosis of plague is mandatory to start prompt antibiotic treatment and prevent severe complications that can lead to death. However, diagnosis remains a challenge, as most human cases appear in remote areas where access to the health system is limited. Diagnosis usually relies on the isolation and culturing of the bacteria on agar plates from a clinical sample, which can be either bubo aspirates, respiratory tract specimens (i.e., sputum), blood, pharyngeal swabs, or urine (12). Y. pestis can grow on rich BHIA; however, MacConkey agar and CIN medium are suggested (11) to limit the growth of contaminant bacteria from respiratory tract, pharyngeal, and sputum specimens. We have previously reported (7) on a selective medium named BIN, which is supplemented with Irgasan, cholate salts, crystal violet, and nystatin; thus, it is preferable for the isolation of Y. pestis from complex specimens, such as respiratory tract, pharyngeal, or environmental samples. However, the preparation of this medium is tedious. Moreover, BIN is based on the use of the nutrient-rich medium BHIA, which typically contains animal tissue infusions and is not a defined medium. Different batches of BHIA offer differential growth support, which is implicated in BIN medium variation. To overcome these obstacles, we have developed an improved BIN medium, with growth properties that are not BHIA batch dependent and that is simple to prepare. By omitting the autoclave step, we achieved better and more homogenous growth support of Y. pestis on BIN plates without losing sterility of the selective medium. Furthermore, we found that preparing a stock solution containing all selective agents, namely, an all-in-one supplement solution, dramatically simplifies the preparation procedure without changing the selective properties on the BIN plates. Moreover, omitting the need to select for a compatible BHIA batch is of great advantage, lowering the overall cost, labor, and time.

The WHO reported that 75% of global plague cases were identified in Madagascar, with an annual incidence of 200 to 700 suspected cases (mainly bubonic plague) (10).

According to the WHO, isolation of Y. pestis from plague sample cultures remains the gold standard (6). However, between 1998 and 2016, the confirmation rate using CIN medium was approximately 27% (5). This low confirmation rate was the result of several factors, such as poorly preserved and contaminated field samples, the presence of contaminants in polymicrobial specimens (e.g., sputum), long delays in transport to the laboratory, and the start of antibiotic treatment before sample collection (5). In addition, the lack of a highly selective medium makes the recovery of Y. pestis difficult. In such cases, the use of an improved culture medium for optimal isolation of Y. pestis is required.

In this study, the improved BIN medium was assessed on more than 50 biological specimens collected from suspected Malagasy plague patients, either retrospectively or prospectively, and compared to the CIN medium routinely used at the CLP plague unit (IPM). We found that the BIN medium allowed the recovery of 8 Y. pestis strains from specimens that tested negative on CIN medium. The performance of the BIN medium is probably due to the omission of cefsulodin and novobiocin, which are in the CIN medium. Indeed, these antibiotics are known to be active against Pseudomonas infection and prevent the growth of Gram-positive bacteria. The concentrations of these antibiotics used in the CIN medium may also inhibit the growth of Gram-negative bacteria and may hamper the growth of Y. pestis if present in very low quantities in biological samples (7).

Overall, BIN was superior to the CIN medium for Y. pestis isolation with respect to buboes and postmortem specimens. This outcome may be caused by the growth-supportive characteristic of BIN agar and its antimicrobial and antifungal components. Indeed, for these primary specimens, bubo pus is considered to be a pure environment for Y. pestis, and the corpse of a plague victim contains a high level of Y. pestis. However, five of eight specimens positive on BIN medium had a high contaminant background on CIN medium, which may suggest that the additional antimicrobials (cefsulodin and novobiocin) in the CIN medium do not prevent the growth of other pathogens and therefore have an impact on its selectivity. However, notably, the 3 remaining specimens that were positive on BIN medium both exhibited contaminants and characteristic colonies of Y. pestis, which can be explained by the fact that the lack of antimicrobials in the BIN medium can result in reduced selectivity to other competitive agents (7). Although more Y. pestis strains have been recovered from the BIN medium compared to the CIN medium, this capability may have been influenced by the type of specimens used. Indeed, only 2 sputum specimens collected from pneumonic plague patients were available for the evaluation of the improved BIN medium, preventing us from evaluating its performance on such complex specimens.

In the public health context, the use of appropriate and efficient medium for Y. pestis recovery is of great value, since the isolated strain allows the surveillance of antibiotic sensitivity of Y. pestis. Indeed, resistant Y. pestis strains have already been isolated in Madagascar. A multidrug-resistant (MDR) Y. pestis (13) and a streptomycin-resistant Y. pestis strain (14) were both isolated in 1995. Resistant Y. pestis strains may represent a threat to the exposed population if they spread in the environment and when the first-line recommended antibiotic (streptomycin) is no longer available. Fortunately, a clinical study comparing the efficiency of ciprofloxacin alone (an alternative treatment for plague) versus streptomycin-ciprofloxacin (the first-line treatment regimen for bubonic plague in Madagascar) is ongoing in multiple study sites in Madagascar (15).

In conclusion, the improved BIN agar is a simple to prepare, highly selective means for Y. pestis isolation and is well suited for remote areas where plague is endemic. All-in-one stocks of selective supplements can be prepared in central laboratories and distributed. Thus, we recommend the use of this medium for the selective isolation of Y. pestis in areas where plague is endemic.
